# Effect of dynamic neuromuscular stabilization on balance and trunk function in people with multiple sclerosis: protocol for a randomized control trial

**DOI:** 10.1186/s13063-022-06015-3

**Published:** 2022-01-21

**Authors:** Laleh Abadi Marand, Shohreh Noorizadeh Dehkordi, Mahtab Roohi-Azizi, Mehdi Dadgoo

**Affiliations:** 1 Rehabilitation Research Center, Department of Physiotherapy, School of Rehabilitation sciences, Iran University of Medical Sciences, Tehran, Iran; 2 Rehabilitation Research Center, Department of Basic Sciences in Rehabilitation, School of Rehabilitation Sciences, Iran University of Medical Sciences, Tehran, Iran

**Keywords:** Multiple sclerosis, Balance, Falling, Spasticity, Exercises, Dynamic neuromuscular stabilization, Core stability

## Abstract

**Background:**

Multiple sclerosis is a chronic and disabling neurological disease among young people. One of the major complaints in patients with multiple sclerosis (PWMS) is falling. There are a number of factors that risk factors for falling, including balance disorder and spasticity. Core stability (CS) exercises such as trunk muscle strengthening exercises can improve balance and mobility and reduce falling. Dynamic neuromuscular stabilization (DNS) exercise is a new functional rehabilitation strategy that optimizes motor function based on the principles of developmental kinesiology. This trial will evaluate the effectiveness of DNS in comparison to CS on balance, spasticity, and falling in PWMS.

**Methods:**

A total of 64 PWMS, between 30 and 50 years old and expanded disability status scale (EDSS) between 2 to 5, will be recruited from neurophysiotherapy clinic, Faculty of Rehabilitation Sciences, Iran University of Medical Sciences to participate in this 2-armed parallel study. Participants will be randomly divided into two groups to receive CS exercise or DNS exercise. All participants will receive exercise treatment for 15 sessions during a period of 5 weeks (3 sessions per week). Primary outcome measures will be balance. Falling rate, fear of falling, patient mobility, as well as spasticity, will be measured as secondary outcomes. All outcome measures will be measured at baseline, the day after the completion of the 15th session, and after 17 weeks.

**Discussion:**

Dynamic neurostabilization exercises utilize the subconscious stimulation of special zones to reflexively mediate the diaphragm and other core stabilization muscles, which is extremely effective for individuals with reduced somatosensory or movement awareness. Findings from the proposed study are expected to benefit the knowledge base of the physiotherapist, and it can be a good alternative for the rehabilitation program and even reduce medication use in patients with multiple sclerosis. These exercises are easy to understand and applicable for these patients and their partners as well.

**Trial registration:**

The trial was registered in the Iran registry organization with code IRCT20140222016680N5 and was approved on April 7^th^, 2020. Address: IRCT administration team, Central Library Building, Iran University Campus, Hemmat Freeway, next to Milad tower, Tehran, Iran. postal code:14496-14535.

## Background

Multiple sclerosis is a chronic neurological disease and the most important cause of non-traumatic disability among young people [1]. Among the wide range of symptoms associated with multiple sclerosis, falling is one of the major complaints and a symptom experienced by more than 50% of PWMS [2]. Falling causes pain, injury and fracture, and fear of falling [3]. There are a number of risk factors for falling in PWMS, including balance disorders, spasticity, and cognitive impairments [4]. Living environment, using cane, fatigue, decreased muscle endurance, heat sensitivity, and urinary incontinence are the other risk factors for falling in PWMS [5, 6]. Patients who have a falling history walk more slowly and cautiously, and cannot correctly push their feet off the ground [7].

Balance impairment is the most common risk factor for falling in PWMS with any degree of disability [8, 9]. Individuals with multiple sclerosis have different strategies to maintain balance in comparison to healthy individuals [10, 11]. They tend to use more compensatory postural adjustments and fewer anticipatory postural adjustments [12]. Lack of trunk control and limited trunk muscle activities is some of the most important causes of balance disorders in PWMS. These patients have delayed onset times for trunk muscles, which are correlated with poor sitting balance. They have been found to have less trunk stability during arm movements compared to healthy subjects [13]. As a result, they have reduced trunk dissociation between the upper and lower trunk, and between the trunk and lower extremity, which is one of the key factors for postural control impairment [14].

Spasticity is another common disorder in PWMS that leads to falling [15]. Spasticity is said to be an annoying and disruptive factor in patient's daily living [16]. It increases the likelihood of falling by increasing energy consumption, fatigue, and disturbing the patient's sleep [15]. Additionally, spasticity decreases the quality of life and causes pain and disability [16, 17]. According to the Lewit and Ryerson approach, disruption of the muscle length-tension relationship, postural instability, and impairment of muscle activity pattern cause high tonocity in lower limb muscles [18].

Some neurologic disorders rather than multiple sclerosis such as stroke, and cerebellar palsy experience high tonocity in lower limbs. Evidence shows that these patients have less trunk control and, as a result, less capacity for selective lower limb movements [18, 19]. However, studies conducted on trunk control in PWMS are limited. It has been shown that the trunk position affects the biomechanics of the lower extremities [20]. Trunk control is the strongest predictor for gait capacity in lower extremity spasticity [19], and there is a high positive correlation between trunk control and balance in patients with spastic limbs such as cerebral palsy [21].

Various studies have investigated the effect of trunk muscle training on reducing balance disorders and falling in PWMS [22–24]. Core stability (CS) training is a controlled form of exercise using the body stabilizing muscles, with emphasis on the deep abdominal muscles [25]. These muscle groups are thought to contribute to trunk stability and optimal lumbar-pelvic stabilization needed for daily activities [26]. CS training is recommended by international multiple sclerosis association guidelines [27] and has been commonly used for PWMS. Although CS exercises can improve balance and mobility, reduce falling rate and fear of falling in PWMS, they have no emphasis on trunk muscle timing and joint positioning [22–24]. Moreover, the combination of spinal stabilization with a dyaphragmatic breathing pattern has been missed in CS training. Dynamic neuromuscular stabilization (DNS) exercises are a new functional rehabilitation strategy that optimizes motor function based on the principles of developmental kinesiology [28]. It is based on the pattern of human motor development in the early years of life that controls posture, maintains it against gravity, and activates muscles purposefully [29, 30]. One of the most fundamental purposes of the DNS approach is to support joints and all segments into a functionally central position, at timing muscle coordination [29]. In DNS, the individual must maintain the intra-abdominal pressure and perform the locomotive movement voluntarily [28]. According to the evidence, DNS is an effective protocol to significantly improve respiratory function [31], and reduce forward head posture [32]. It was effective in standing, walking, and jumping in patients with spastic diplegic cerebellar palsy [33], and in addition, DNS can improve trunk function, balance, and fear of falling in individuals with hemiparetic stroke [34]. Thus, we hypothesize that by establishing a normal postural alignment and proper trunk muscle activation pattern by DNS in PWMS, balance may be improved and the tone of lower limbs can be reduced. The aim of this study is to determine the effects of DNS exercises on balance, trunk function, falling, and spasticity, and compare them with CS exercises.

## Methods and design

### Trial design

This study is a two parallel armed, parallel groups, superiority randomized controlled trial, compared DNS exercise, to CS exercises in PWMS. This randomized controlled trial is with one to one allocation in two groups (group 1: DNS exercise, group 2: CS exercise). The time schedule of enrolment, interventions, and assessments is presented in Table 1.

**Table 1:**
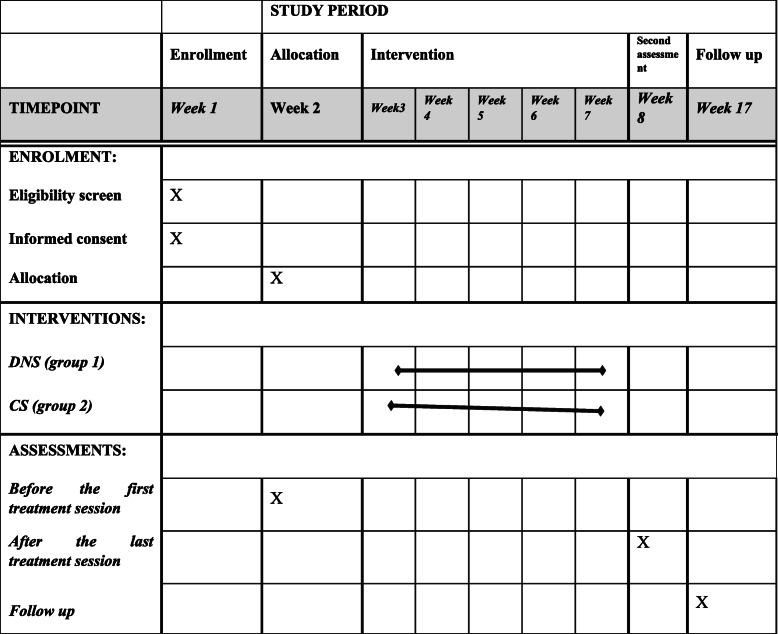
Schedule of enrollment, intervention, and assessment of PWMS

### Participants

Women and men with multiple sclerosis aged between 30 and 50 years with moderate disabilities (EDSS between 2 and 5) will be included in the study from June 2020.

### Inclusion and exclusion criteria

Patients with a minimum score of 21 from the mini-mental scale examination, ability to walk, having sustained falling during the last 3 months, and at least high school graduation to fill out self-declaration questionnaires will be eligible. Patients with other neurological disorders, multiple sclerosis exacerbation during the last month, apraxia (due to inability to learn and practice the exercises), severe spasticity (score above three based on modified Ashworth scale), having a history of major surgery in the lower extremities, explicit postural abnormalities in the spine and lower limbs such as scoliosis and kyphosis and cardiovascular disease will be excluded.

### Recruitment procedures

Recruitment will take place in two steps: firstly, patients who were previously confirmed by neurologists for multiple sclerosis are referred to the neuro-physiotherapy clinic, Faculty of Rehabilitation Sciences, Iran University of Medical Sciences. Secondly, the assessor, who is a researcher, will conduct another screening for inclusion and exclusion criteria, will evaluate outcome measures, and make the final decision regarding the eligibility of the patients the same day; patients are referred to the clinic. If eligible, the patient will be provided with oral and written information about the study. Background information including name, contact number, height and weight, medical history, and medications used will be obtained from participants. Having signed a consent form by the treatment provider, patients will be randomized to one of the two treatment groups. and the assessment will be performed the same day of screening, in the physiotherapy evaluation laboratory in the School of Rehabilitation Sciences at the Iran University of Medical Sciences, Tehran.

One or two days later treatment sessions will be started in participants’ houses for 5 weeks (three times a week) by a treatment practitioner who is a physiotherapist with five years of experience in the field of neuro-physiotherapy. Because of the COVID-19 pandemic, all treatment sessions will be held in patient’s houses and the patients will come to physiotherapy evaluation laboratory for three times of assessment (baseline, one or two days after 15th session and 17 weeks after randomization day). All treatment sessions are free for all participants and the cost of any travel to the evaluation laboratory for assessment will be borne by the research team.

### Randomization

The participants, with the permuted block randomization method, will be randomized to dynamic neuromuscular stabilization exercises or core stability exercises by an independent researcher not involved in outcome assessments. Sixteen quadruple blocks will be produced using the site www.sealedenvelope.com. In order to apply concealment in the randomization process, unique codes will be used on the envelopes in which the type of exercises is specified by an analyzer.

### Concealment

For concealment in the randomization process, a unique code will be used on each envelope with the group number (1 or 2) specified inside which will be done by a blind analyzer.

### Implementation

Allocation sequence, participant enrollment, and participant assignment to treatment groups will be performed by an independent person who is not involved in assessments, intervention, and any other part of the research.

### Blinding

In this study, the assessor who evaluates the outcome of the study will be blind to the allocation of the treatment groups and doesn’t provide intervention. After each assessment session, she will enter all data electronically. It may be done in Rehabilitation Research Center, Department of Physiotherapy, where the data will have gathered. Additionally, the analyzer who is independent from the research team and is blind to participant’s allocation will monitor data of the study after trial termination and analyze data.

### Sample size calculation

This study, for the first time, compares DNS exercises with CS exercises in PWMS. Due to the lack of previous studies and considering the main goal of study as comparing two groups the day after the completion of the 15th sessions, the Cohen standardized effect size for comparing means of two independent groups was used in this study to calculate the sample size. Considering the probability of the first type error of 5%, the power of 80%, the standard Cohen’s effect size of 0.8, and using G-Power software the sample size was calculated as 26 subjects in each group which increased to 32 subjects to assume the 20% of dropout rate. Another power calculation will be performed at the end of the trial to assure the sufficiency of the sample size.

### Interventions

At first, necessary explanations about the importance of exercise therapy in PWMS will be given to the participants. In both groups, exercise sessions will be delivered three times a week, over a course of 5 weeks, and the duration of each session will be 60 to 75 min. All 15 sessions will be supervised by the treatment practitioner at the participant’s houses individually due to COVID-19. In addition, the participants or their companions are required to make the home environment as safe as possible to minimize the likelihood of falling.

In two pamphlets, both groups will be given the exercises (DNS and CS) with the description required to do their exercises on days when the treatment practitioner is not present. In order to remind exercises to the participants, a reminder will be set on their cellphones. In addition, the participants will be instructed not to do any other exercises, nor receive other types of physiotherapy treatment during 5 weeks’ treatment sessions. If female participants get pregnant during the treatment plan or any fracture accrued during treatment sessions, they will be excluded.

Four barriers to exercise in PWMS are anticipated. The first is fatigue. Rest intervals are taken between specified exercises depending on one’s condition. Participants will be highly recommended to stop any exercise movement when they feel a lack of energy and any fatigue signs which are important in PWMS will be discontinued treatment sessions. The second is menstruation in women. The treatment session will be canceled on the first day of menstruation. The third is the time of the treatment session. The exercises will be performed in the morning or evening based on the participant’s comfort and level of energy. The fourth is medication. Treatment sessions will take place 48 h after the participant’s multiple sclerosis-related medications.

### Dynamic neuromuscular stabilization exercises (group 1)

At first, the person learns the correct posture in the sitting and standing position in front of a mirror. They recognize their abdominal muscles and learn how to contract them. Then, the treatment practitioner will teach participants diaphragmatic breathing.

#### Diaphragmatic breathing training

It is recommended to sit in an erect position. The shoulders and neck should be completely relaxing, and the upper abdominal muscles should not be active. The person is asked to push the anterior and lateral walls of the abdomen out during the inhale, and perform eccentric contraction of the abdominal muscles. During exhale, they need to maintain intra-abdominal pressure with abdominal muscle contraction. Exhale time should be longer than the inhale time [29].

During each step and in every situation, diaphragmatic breathing is emphasized so that the patient follows this type of breathing in each prescribed exercise.

The exercises will be performed in four positions: supine, prone, sitting, and standing as follows:

#### Prone position

One turns his head to one side, bending the hand and leg on the same side, then bending the hand and opposite leg.

The shoulders and elbows in 90–90 position, lifting the chest first from the floor and then getting the elbows closer to each other and bearing weight on elbows. Next, they take forearms off the ground and bear the weight on the palms of the hands, and then take the pelvic off the ground to reach the quadruped position. To separate the upper trunk from the lower trunk, move hips to each side while maintaining the upper trunk [33].

#### Supine position

The thigh and knee are positioned 90–90, and the hips are slightly abducted and externally rotated. The person is then asked to raise their hands to grab their knees first and then their toes. To separate the upper trunk from the lower trunk, the patient is asked to rotate the upper trunk to the left and right (without moving the lower trunk) while the arms are straight in front of the chest. Again to separate the upper trunk from the lower trunk, the lower trunk is rotated to the left and right (without moving the upper trunk) by rotating the bent knees to each side.

Simultaneous rotation of the upper trunk to one side and lower trunk to the other is the next step: changing of position from supine to side lying, with upper and lower limb flexion and adduction and trunk flexion and rotation. Then moving from side-lying to prone position while controlling the speed [33].

#### Sitting position

In long sitting position, participants put their hands out of their knees, slowly bend knees and hips, then with a slight external rotation of the hips, they try to grasp the toes. In an erect sitting position on a chair, the soles of feet on the floor, arms hooked, they rotate trunk to each side. In this position, they bend the trunk to each side to lift an object. Then they lift pelvis on each side while the trunk is fixed [34].

#### Sit to stand transformation

They sit erect on the ground, one knee outward and one knee inward (side sitting). Then, with the help of a ladder, they put the foot of the in-warded knee on the ground to bring the knee forward.

In the knee-forward position, they do the forward/backward and lateral weight shifting and trunk rotation to the sides. Then bring the other leg forward and switch to squat position, and then stand upright [28]. During the first to the fifth week, the number of sets and their repetition will be increased based on one’s performance.

### Core stability exercises (group 2)

#### Abdominal muscles contraction

Before starting, participants need to recognize their abdominal muscles and learn how to contract them. They will be asked to maintain abdominal contraction and normal breathing (squeeze abdomen while inhalation and exhalation) in all exercises [24].

#### Supine position

In supine position, participants bend their knees and put soles of both feet on the ground (crook lying). While the spine is in proper position, they pull their navel inward (squeeze abdomen gently) and maintain this condition for 5 s. Then, they do the same exercise with straight knee position. In crook lying position, first participants take the sole of each foot off the ground alternately, then lift both legs simultaneously and take both knees to the abdomen. As they look at the ceiling, they gently take their shoulders off the ground (curl up). Next, while both feet are on the floor, they gently flex and extend their knees (heel slide). Participants do bridging exercises while they contract their abdominal and gluteal muscles [22].

#### Prone position

At first, they do the abdominal contraction in the prone position. Then, they raise their chest so that they are in prone on elbow position. In the same position, they take the pelvis off the ground, while the knees and forearms are on the ground (semi plank). Next, they raise the pelvis to be in the quadruped position [23].

#### Quadruped position

In this position, they contract abdominal muscles, then they do cat-camel exercise. After that, they raise their hands than their legs alternately. In the same position, participants raise the opposite hand and leg simultaneously [24].

#### Side-lying position

While two knees are bent, they push the elbow on the ground to raise their trunk and pelvic off the ground (semi-side bridge) [22].

#### Sitting position

While sitting on a chair and soles of the feet on the floor, they raise their knees to the abdomen alternately [23].

#### Standing position

As they lean against the wall, they contract abdominal muscles and gently lower the trunk as the knees bend [24].

During these 5 weeks, the number of sets and their repetition will be increased based on one’s performance.

### Outcome measures

All primary and secondary outcome measures will be measured by the blind assessor, who is a physiotherapy PhD student and completed a training course of biomechanical assessment tools, at baseline (a day before the first treatment session), the day after the final treatment session (15th session), and at a follow-up measurement after 17 weeks s. A home-based exercise program will be recommended for 17 weeks s’ follow-up based on their exercise group.

#### Primary outcome measure

Primary outcome measure will be balance measured by the Berg Balance Scale.

##### Berg Balance Scale

Berg Balance Scale is a validated tool to measure a patient’s static and dynamic balance. It consists of 14 items (scores from zero to 56) and is a valid and reliable test for PWMS [35]. Berg Balance Scale measures balance in sitting, standing, and most daily activities (such as sitting up, moving from chair to chair, standing with eyes closed).

#### Secondary outcome measures

Secondary outcome measures will be:

- Trunk function measured by the Trunk Impairment Scale
- Postural stability by the Biodex Balance System
- Falling rate by asking the patient or their partner: “During the last 6 months, how many times have you fallen down?”
- Fear of falling by filling out Activities-specific Balance Confidence questioner.
- Patient mobility by filling out Multiple Sclerosis Walking Scale-12 questioner and measured by Time Up and Go test.
- Spasticity scale by filling out Multiple Sclerosis Spasticity Scale-88 questioner and measured by Modified Ashworth Scale.

##### Trunk Impairment Scale

The Trunk Impairment Scale is a tool to assess trunk function ability in static, dynamic, and coordination conditions, with scores ranging from zero to 23. The scores for all items are summed, and a higher score indicates greater trunk function. In all cases, the person is in the sitting position without leaning back and without using their hands. Trunk Impairment Scale has been validated and tested for reliability in PWMS [36].

##### Postural stability

Biodex Balance System SD, 115 VAC (made in the United States) is a balance system consisting of a balance plate with 20° tilt range and various stability from 1 (least stable) to 12 (most stable). It is a valid and objective instrument to assess balance performance [37]. The study subjects will place their bare feet on the plate. Foot angle and location of the heel of each foot are recorded in the first session so that they will not change in subsequent sessions. The screen is adjusted to fit the individual height. Before starting the test, the individual will be familiarized with test’s performance (postural stability and falling risk tests). Participants will be instructed to keep the black circle on the screen in the middle of the screen. The number of repetitions in each test is set to three (20 s each with 10 s to rest interval). The plate stability starts at 12 and decreases by the end of the test based on each person's ability. Overall stability, anterior-posterior and medial-lateral index, and falling risk index for each individual will be recorded.

##### Activities-specific Balance Confidence

Activities-specific Balance Confidence is a questioner that contains 16 items and the participant rates his or her perceived level of balance confidence for each item. Scores range from 0 to 100 (best score). It is validated for PWMS [38].

##### Multiple Sclerosis Walking Scale-12

The Multiple Sclerosis Walking Scale Questionnaire is a subjective questionnaire with 12 items. It is a valid and reliable scale where participants rate the extent to which multiple sclerosis has limited their walking ability during the past 2 weeks (maximum score =100) [39].

##### Time Up and Go test

Time Up and Go test is a valid and reliable test to measure the mobility of PWMS. The tools needed in this test are a chair with proper height, a backrest, and a stopwatch. The person sits on the chair, and 3 m in front of the person is marked. They will be instructed to get up from the chair as quickly as possible, go up to 3 m, turn around and follow the same path and sit on the chair [40]. The assessor records the time spent with a stopwatch. The test is repeated three times, and the average is recorded.

##### Multiple Sclerosis Spasticity Scale-88

Multiple Sclerosis Spasticity Scale-88 measures patient’s pexperience and perception of the impact of spasticity in Multiple Sclerosis with day-to-day symptoms and during functional activities over the previous 2 weeks. It contains 88 questions to quantify spasticity for a total score and in eight clinically relevant and stand-alone subscales: muscle stiffness, pain and discomfort, muscle spasms, activities of daily living, walking, body movements, emotional health, and social functioning [41]. Summation of each item score will be recorded.

##### Modified Ashworth Scale

Modified Ashworth Scale is the most commonly used clinical measure of spasticity. Its validity has been proven in all muscle groups. In this study, the group of knee extensors and ankle plantar flexors will be evaluated. First, the inactive range of motion of the knee and ankle joints is measured by goniometer. To measure the tone of the knee extensors, the individual sleeps in the side-lying position so that hip and knee joints are in extension and head and trunk in a straight line, and the pelvis is supported from behind. The assessor stands behind the patient, with one hand above the knee outside the thigh, which stabilizes the femur. The other hand is above the ankle. Then, for 1 s (with the word one thousand one), the knee is passively moved from maximal extension to maximal flexion. The grade ranges from 0 to 4. To evaluate the tone of ankle plantar flexors, the person lies in the supine position, hip and knee are in extension, head and trunk in the midline. The assessor stands on the test side with one hand on the knee (to keep the lower limb fixed) and the other hand under the foot. He/she then lifts the ankle from maximum plantar flexion to maximal dorsiflexion within one second [42].

### Data analysis

SPSS version 22 will be used for data analysis. We will perform an intention-to-treat analysis for dropout participants during treatment sessions and in follow-up assessment sessions. Conditional mean imputation was performed. Mean (standard deviation) or median (first quartile and third quartile) will be used to describe the quantitative variables according to the distribution of variables, and for categorical variables, frequency (percentages) will be used. Graphical methods, numerical indices, and Shapiro-Wilk’s tests will be used to check the normality of different measures.

To compare quantitative demographic variables between the two groups, an independent *t*-test or nonparametric test, Mann Whitney *U*-test will be used. Chi-squared or Fisher’s tests will be used to compare the categorical demographic variables between the two groups.

A separate regression analysis will be done at each follow-up point (i.e. the day after the completion of the 15th session (T2), and after 17 weeks follow-up (T3) each one including group and baseline (T1) as a covariate. The T2 and T3 follow-up point will be considered primary and secondary, respectively. A non-parametric analysis would be a Mann-Whitney *U*-test of the differences (follow-up minus baseline). A 2-tailed *P* < 0.05 will be considered significant.

## Discussion

Findings from the proposed study are expected to benefit the knowledge base of the physiotherapist, and health care professional. If these exercises can reduce fall rates by lowering spasticity and improving balance, it can be a good alternative for the rehabilitation program and even reduce medication use in PWMS. Although there is various type of exercises for PWMS, they are mostly focused on balance specialized and lower limb muscles strengthening exercises which can in the short time make a difference and may not benefit the patient for a long time. As mentioned before, trunk muscle exercises such as CS exercises have been used in the rehabilitation of PWMS recently. CS exercises can reduce the falling rate and fear of falling and improve balance and mobility in PWMS [22–24]. However, DNS was developed based on neurodevelopmental kinesiology and reflex-mediated core stabilization concepts. DNS utilizes the subconscious stimulation of special zones (chest zones) to reflexively mediate the diaphragm and other core stabilization muscles, which is extremely effective for individuals with reduced somatosensory or movement awareness [30]. Despite the use of DNS in some disorders [31–34], there is limited evidence of the effectiveness of this method in PWMS. So, the current study’s aim is to compare the effect of DNS to CS exercises in balance, falling, and spasticity in PWMS.

Strengths of our study design include that is the first randomized control trial on DNS exercise effects on PWMS. A 17-week follow-up is predicted after exercise sessions. In addition, both groups of exercise packages (DNS and CS) are easy to understand and applicable for PWMS and their partners as well. We also recognize the possible limitations of the study. The inclusion criteria, preclude the majority of participants as we limit age range and EDSS. Consequently, we might encounter difficulties in finding desired participants and we may have some dropouts.

## Trial status

Enrollment of participants into this study started in June 2020. Target enrollment for this study is 64 participants. Trial ID is 46429 which was approved in 7th of April 2020. It was updated for the first time on 22nd January 2020, the second time on the 31st of August 2021, and the last time on 18th November 2021.

## Data Availability

The datasets used and/or analyzed during the current study are available from the corresponding author on reasonable request. All data will be entered automatically by the assessor. This will be done at the Rehabilitation Research Center. A special folder will be provided for each person participating in the study, which will include the questionnaires they have filled out as well as the results of all other assessments. The name of the folder for each participant will be a special code that will be dedicated to each participant. These folders will be stored in a secure and accessible place and manner. All required processes will also be done to promote data quality (e.g. double data entry; range checks for data values).

## Abbreviations

PWMS: Patients with multiple sclerosis
CS: Core stability
DNS: Dynamic neuromuscular stabilization
EDSS: Expanded Disability Status Scale
